# Management of Severe Epistaxis during Pregnancy: A Case Report and Review of the Literature

**DOI:** 10.1155/2019/5825309

**Published:** 2019-01-20

**Authors:** Maria Grazia Piccioni, Martina Derme, Laura Salerno, Elisa Morrocchi, Francesco Pecorini, Maria Grazia Porpora, Roberto Brunelli

**Affiliations:** Department of Gynecological, Obstetrical and Urological Sciences, University of Rome “Sapienza”, Viale del Policlinico, 155, 00161 Rome, Italy

## Abstract

**Background:**

Epistaxis is a common problem during pregnancy. Few cases of severe epistaxis, not associated with nasal lesions or clotting disorders, were described in the literature. We reported a case of severe epistaxis in a pregnant patient, exploring all the different possible management options.

**Case:**

A 33-year-old primigravida, who was 38 weeks pregnant, presented with spontaneous severe left-sided epistaxis. Her blood pressure was into normal ranges. Clotting disorders and nasal lesions were excluded. The patient clinical worsening, due to severe anemia, and the failure of conservative treatment have imposed an emergency caesarean section, with an immediate resolution of the nasal bleeding.

**Conclusion:**

Treatment of severe epistaxis must always consider conservative measures first-line with early recourse to otolaryngologist. In general, delivery of the fetus is considered curative.

## 1. Introduction

Epistaxis is a common problem during pregnancy, due to an increased nasal mucosa vascularity. The prevalence in pregnant women is 20.3% compared with 6.2% in nonpregnant ones [1]. Large volume epistaxis is rare for patients without preexisting risk factors or conditions, such as the use of anticoagulants or blood clotting disorders.

Few cases of severe epistaxis during pregnancy, not associated with nasal lesions or clotting disorders, were described in the literature, demonstrating a lack of familiarity regarding the appropriate management options in these clinical conditions.

We report a case of severe, prolonged epistaxis in a pregnant patient, during the third trimester, with no clear risk factors. We explored the different possible management options in this challenging case and we assume our experience may help in future similarly clinical situations.

## 2. Case Presentation

A 33-year-old primigravida, who was 38 weeks pregnant, presented with spontaneous severe left-sided epistaxis. Her first episode had started the previous week, with about seven-eight episodes a day. Her medical history was unremarkable. She had no personal or family history of bleeding tendencies, and she was not taking any regular medications. Her blood pressure was into normal ranges. She reported no previous episodes of epistaxis in her life. Routine blood tests were normal during the pregnancy.

We tried to control the bleeding first by administrating intravenous (IV) tranexamic acid, without resolution. So, we contacted the otolaryngologist, who performed an endoscopy, showing a left nasal floor bleeding varix. He decided for an anterior nasal packing: he inserted a tampon carefully along the floor of the left nostril, where it expanded on contact with blood. After the nasal tampon was been inserted, the otolaryngologist wetted it with a small amount of topical vasoconstrictor in order to hasten effectiveness. This procedure was repeated three times, inserting a total of six tampons (four in left nostril and two in the right one). Nevertheless, this conservative management of epistaxis failed. Within 4 hours of admission, patient haemoglobin had dropped from 12.5 to 7 mg/dl and she had a further bleed from the left nostril. The otolaryngologist did not consider a posterior nasal packing because the endoscopy showed an anterior bleeding site. A new endoscopy to locate the exact site of bleeding for direct cauterization was not indicated in acute setting due to vascular congestion and mucosal oedema. Patient clotting studies were within the normal range. A blood transfusion was required, using two packed red blood cells (PRBCs). The patient also started antibiotic therapy with IV Cephazolin 2 g every 8 hours. Cardiotocography (CTG), biophysical profile, and fetal Doppler demonstrated fetal well-being.

During her second day of admission, repeated blood tests showed that her haemoglobin remained persistently low at 7.5 g/dl, despite the recent blood transfusion. The patient became tachycardic (rate 157 bpm), tachypnoeic (22 breaths per minute), and asthenic.

After accurate counselling with the patient and considering the failure of conservative treatment, we thereby decided for a surgical management of pregnancy. The patient delivered a healthy baby girl weighing 3.9 kg. The execution of caesarean section was followed by an immediate resolution of the nasal bleeding.

We discharged the patient from the hospital with nasal packing, in order to ensure the formation of an adequate clot. Five days later, the otolaryngologist performed an endoscopy to locate the exact site of bleeding for direct cauterization. The patient experienced no other episodes of epistaxis.

## 3. Discussion

Epistaxis in pregnancy is common but the vast majority of cases do not require medical attention. The prevalence of epistaxis in pregnant women is more than three times that in nonpregnant ones [1].

Several conditions predispose to epistaxis during pregnancy. In particular, the elevated oestrogen levels increase the vascularity of the nasal mucosa [10], which may potentiate and prolong the bleeding. Progesterone causes an increase in blood volume, which may exacerbate both vascular congestion and hence bleeding, and may mask blood loss in the event of severe epistaxis, due to apparently effective cardiovascular compensation [11]. Placental growth hormone has systemic effects, including vasodilation [11]. Indirect hormonal effects include vascular inflammatory and immunological changes that may predispose to nasal hypersensitivity and hence to problems such as nasal granuloma gravidarum [11]. In general, delivery or fetal death causes immediate cessation of the nasal bleeding, because some of the underlying factors, such as congestion and hyperemia, disappear.

Few cases of severe epistaxis during pregnancy were described in literature [2–8, 12] (Table 1). We excluded cases of epistaxis associated with nasal lesions, like granuloma gravidarum [9] and nasal polyp [13], or clotting disorders [14].

Treatment of severe epistaxis must always consider conservative measures first-line, like IV tranexamic acid administration, anterior packing and bipolar cautery. If conservative treatment fails, two radical treatments have to be considered: the one is surgical, in the form of vessel ligation, and the other is obstetrical and is termination of the pregnancy.

In our case, the patient clinical worsening and the failure of conservative treatment imposed an emergency caesarean section. The cervix was unfavorable for easy induction and a long induction of labor was considered contraindicated for this patient. Valsalva maneuvers could also aggravate the bleeding during labor, increasing the risk of fetal hypoxia. The decision to deliver was also influenced by the gestational age; in fact in the case of a preterm pregnancy, when maternal and fetal conditions are good, a conservative management is preferred, in order to avoid the possible risks associated with preterm birth.

Fetal anemia is a well-known cause of antenatal fetal distress. The case report by Braithwaite JM et al. [5] demonstrated that rapidly developing severe maternal anemia, due to recurrent blood loss of nonplacental origin, even in the absence of maternal hypotension, can cause fetal distress. Severe epistaxis is potentially life-threatening to both mother and fetus.

This case highlights the importance of early recourse to ear, nose, and throat (ENT) referral, when epistaxis is unresponsive to simple measures. In general, when nasal lesions and clotting disorders cannot be identified, fetal delivery is considered curative, showing that hormonal changes during pregnancy may lead to significant alterations of nasal physiology, with oestrogen causing vascular congestion, mucosal oedema, and rhinitis, known as the “rhinitis of pregnancy”. Moreover, pregnancy is associated with significant anatomic and physiologic remodeling of the cardiovascular system. Starting at 6-8 weeks of gestation and peaking at 32 weeks, maternal blood volume increases by 40–50% above nonpregnant volumes [15, 16]. Termination of pregnancy resolves hypervolemia and hormonal changes; in fact in all the cases reported in Table 1, we can observe a nasal bleeding resolution after delivery.

## Figures and Tables

**Table 1:** Cases of severe epistaxis during pregnancy not associated with nasal lesion or clotting disorders.

Author/year	Number of patients	Management of epistaxis	Management of pregnancy
Green L K 1974 [2]	1	Local pressure; nasal packing	Emergency caesarean section

El Goulli M 1979 [3]	1	Nasal packing	Vaginal delivery

Howard DJ 1985 [4]	1	Nasal packing; bipolar diathermy; external carotid artery ligation; nasal balloon	Emergency caesarean section

Braithwaite J M [5]	1	Nasal packing; nasal balloon	Emergency caesarean section

Cooley 2002 [6]	1	Nasal packing; nasal balloon	Emergency caesarean section

Hardy 2008 [7]	1	Nasal packing; bipolar cautery; artery ligation	Vaginal delivery

Cornthwaite K 2013 [8]	1	Nasal packing; bipolar diathermy	Elective caesarean section

Crunkhorn RE 2014 [9]	1	Nasal packing; sphenopalatine artery (SPA) ligation; bipolar cautery; bipolar diathermy	Elective caesarean section

Our case 2018	1	IV*∗* tranexamic acid nasal packing; bipolar cautery	Emergency caesarean section

Footnotes: *∗* intravenous.

## References

[1] Dugan-Kim M., Connell S., Stika C., Wong C. A., Gossett D. R. (2009). Epistaxis of pregnancy and association with postpartum hemorrhage. *Obstetrics & Gynecology*.

[2] Green L. K., Green R. S., Harris R. E. (1974). Life-threatening epistaxis associated with pregnancy. *American Journal of Obstetrics & Gynecology*.

[3] El Goulli M., Chelli M. (1979). Severe epistaxis during pregnancy. A case history. *Journal de Gynécologie Obstétrique et Biologie de la Reproduction*.

[4] Howard D. J. (1985). Life-threatening epistaxis in pregnancy. *The Journal of Laryngology & Otology*.

[5] Braithwaite J. M., Economides D. L. (1995). Severe recurrent epistaxis causing antepartum fetal distress. *International Journal of Gynecology and Obstetrics*.

[6] Hardy J. J., Connolly C. M., Weir C. J. (2008). Epistaxis in pregnancy - not to be sniffed at!. *International Journal of Obstetric Anesthesia*.

[7] Cornthwaite K., Varadharajan K., Oyarzabal M., Watson H. (2013). Management of prolonged epistaxis in pregnancy: Case report. *The Journal of Laryngology & Otology*.

[8] Crunkhorn R. E. M., Mitchell-Innes A., Muzaffar J. (2014). Torrential epistaxis in the third trimester: A management conundrum. *BMJ Case Reports*.

[9] Noorizan Y., Salina H. (2010). Nasal septal haemangioma in pregnancy. *Medical Journal of Malaysia*.

[10] Goldstein G., Govindaraj S. (2012). Rhinologic issues in pregnancy. *Allergy & Rhinology*.

[11] Sobol S. E., Frenkiel S., Nachtigal D., Wiener D., Teblum C. (2001). Clinical manifestations of sinonasal pathology during pregnancy. *Journal of Otolaryngology*.

[12] Cooley S. M., Geary M., O'Connell M. P., Keane D. P. (2002). Hypovolaemic shock secondary to epistaxis in pregnancy. *Journal of Obstetrics & Gynaecology*.

[13] Scott P. M. J., Van Hasselt A. (1999). Case report of a bleeding nasal polyp during pregnancy. *Ear, Nose & Throat Journal*.

[14] Aynağolu G., Durdağ G. D., Özmen B., Söylemez F. (2010). Successful treatment of hereditary factor VII deficiency presented for the first time with epistaxis in pregnancy: A case report. *The Journal of Maternal-Fetal and Neonatal Medicine*.

[15] Hytten F. E., Paintin D. B. (1963). Increase in plasma volume during normal pregnancy. *Obstetrical & Gynecological Survey*.

[16] Costantine M. M. (2014). Physiologic and pharmacokinetic changes in pregnancy. *Frontiers in Pharmacology*.

